# Vitamin D in Children’s Health

**DOI:** 10.3390/children1020208

**Published:** 2014-09-12

**Authors:** Joy A. Weydert

**Affiliations:** Department of Pediatrics, University of Kansas Medical Center, 3901 Rainbow Blvd., MS 4004 Kansas City, KS 66160, USA; E-Mail: jweydert@kumc.edu; Tel.: +1-913-588-6364; Fax: +1-913-588-6338

**Keywords:** vitamin D, immune system, cancer, pain, mental health, autism, pregnancy, economic impact

## Abstract

Knowledge of vitamin D in the health of children has grown greatly over the years, extending past the importance for calcium homeostasis and bone growth. There is growing recognition of the role vitamin D plays in health impacting the innate immune system to prevent infections and the adaptive immune system to modulate autoimmunity. Other studies are starting to reveal the neurohormonal effects of vitamin D on brain development and behavior, with a link to mental health disorders. Many of these effects start well before the birth of the child, so it is important that each pregnant woman be assessed for vitamin D deficiency and supplemented for the best possible health outcome of the child. It is recommended that targeting a 25(OH)D level of 40–70 ng/mL for each individual would provide optimal health benefits and reduce health care costs. Current recommended doses of vitamin D supplementation fall short of what is needed to obtain ideal serum levels. A vitamin D supplementation program to prevent disease, much like the current vaccination program, could potentially have a dramatic impact on overall health worldwide.

## 1. Introduction

The role of vitamin D has been widely publicized in the popular press promoting health benefits beyond that of bone mineralization. Some of the claims state that vitamin D reduces the incidence of cancer, prevents viral illnesses, treats musculoskeletal pain and stabilizes mood disorders such as depression. There has also been increased interest in the scientific community to study vitamin D both at the basic science and clinical levels to address these claims plus others. From Pub Med [1] greater than 60,000 citations are available related to vitamin D alone. As a result, a wealth of information has been produced that adds to our understanding of how this hormone affects virtually every cell in the body.

In this article, basic vitamin D biochemistry will be reviewed to present the current understanding of its action on various systems throughout the body. Additionally, literature from basic science and clinical studies on vitamin D in relation to current disease states will be presented. Lastly, there will be a discussion on the potential economic impact on health care if Vitamin D levels were optimized for all individuals starting before birth.

## 2. Discussion

### 2.1. Vitamin D Biochemistry

**Figure 1:**
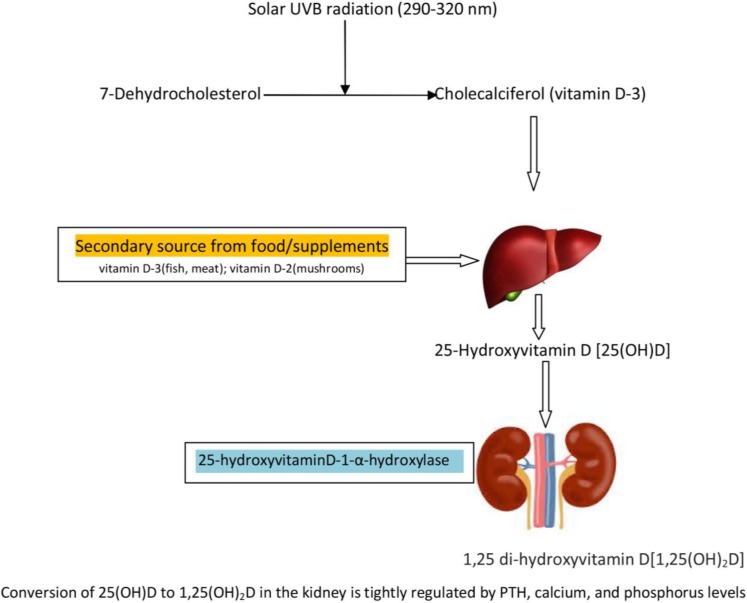
Skin—Principal Source of Vitamin D Production.

In contrast to its name, vitamin D is not a vitamin, but rather a steroid hormone. Vitamins are anti-oxidants or co-factors in enzymatic reaction that primarily come from food. Steroid hormones, on the other hand, regulate gene expression—turning on and off protein production as the body requires. Vitamin D is produced by activation of plant and animal sterol fractions, phytosterol and cholesterol respectively, by sunlight (Figure 1). Plant sterols activated by UVB irradiation produce vitamin D-2. In animals and humans, 7-dehydrocholesterol, the vitamin D precursor found primarily in the epidermal layer of the skin, is activated by sunlight to produce vitamin D-3 and is bound to vitamin D binding protein (VBP). This is transported to the liver where it is rapidly hydroxylated by vitamin D-25-hydroxylase to form 25-hydroxyvitamin D [25(OH)D], the major circulating form of vitamin D. This is considered a pro-hormone with no innate hormone activity in this state [2]. Through further hydroxylation by the enzyme 25-hydroxyvitamin D-1-α-hydroxylase, 25 (OH)D is converted into the biologically active form, 1,25 di-hydroxyvitamin D [1,25(OH)2D]. 1,25 di-hydroxyvitamin D regulates more than 200 different genes, directly or indirectly, by binding to vitamin D nuclear hormone receptors (VDR) that drive a wide variety of biological processes. Most of the conversion of 25 (OH)D to 1,25(OH)_2_D occurs in the kidney and is tightly regulated by parathyroid hormone (PTH), calcium, and phosphorus levels. In this activated state, vitamin D has classic endocrine effects and regulates serum calcium and bone metabolism [3]. The conversion to 1,25(OH)_2_D also occurs in various tissues such as brain, breast, and skin, and in monocytes and macrophages. This local production of 1,25(OH)_2_D regulates cell proliferation, differentiation, and apoptosis as well as augment immune function at those sites [4]. Through this mechanism, vitamin D affects cells directly by its autocrine and paracrine functions and is under autonomous control [5]. VDR are found ubiquitously in the nucleus of all tissues and cells of the immune system and can respond to the activated 1,25(OH)_2_D for gene expression at virtually any site in the body. Having these various endocrine and paracrine functions may explain why vitamin D has wide spread effects on various disease processes.

Having adequate levels of 25 (OH)D is crucial for optimal production of activated 1,25(OH)_2_D. Food provides a limited source of Vitamin D (salmon, sardines, tuna, cod-liver oil for Vitamin D3, and egg yolks or shiitake mushrooms for Vitamin D2) therefore diet alone only provides 100 to 200 IU of vitamin D per day. Exposure to sunlight, in contrast, produces 10,000 to 20,000 IU when 30% of the body surface area is exposed to sunlight 15 to 30 min a day [6]. Sunlight produces UVA, UVB, and UVC rays, each with different skin penetrations and biological actions. It is only a very narrow band of UVB rays (290–320 nm) that activates the 7-dehydrocholesterol in the epidermis. The UVB rays that reach the earth’s surface is directly affected by the zenith angle of the sun. Very little UVB reaches the earth’s surface in the early morning or late afternoon hours of the day due to the oblique nature of the sun's rays. UVB rays that are most efficient in producing vitamin D are available when the sun is most perpendicular to the earth’s surface—between 10 AM and 3 PM. The zenith angle is also affected by season and latitude. In the northern and southern hemispheres, beyond the 33° latitude, the zenith angle is at its minimum during the winter months with virtually no vitamin D production possible [7].

Besides season, time of day, and latitude, UVB exposure is limited by other factors. Sunscreens reduce vitamin D production by 95% (SPF 8) to 99% (SPF 15). Institutionalized persons in prisons, schools, nursing homes, or hospitals get very little direct sun exposure. Dark skinned persons require 10 to 15 times the same sun exposure to produce equivalent amounts of vitamin D than in light skinned persons. Melanin absorbs UV radiation and competes for UVB photons that are needed for vitamin D production. [8] Air pollution and clothing that covers the entire body, as required in some cultures, also reduces UVB exposure. Additionally, obesity impacts the amount of circulating vitamin D that is produced in the skin as subcutaneous fat sequesters the synthesized vitamin D in its cells making it unavailable for conversion to 1,25(OH)_2_ D [2].

Certain medications and medical conditions can also affect the amount of circulating vitamin D. Drugs that are dependent on the cytochrome P-450 system for metabolism, such as phenobarbital, valproic acid, and ketoconazole, compete with Vitamin D for this pathway. Drug-vitamin studies have shown a decrease in vitamin D levels with use of these medications. Malabsorption disorders, such as cystic fibrosis or Crohn’s disease, liver disease, and kidney disease, also affect vitamin D levels and utilization [4].

### 2.2. Measurement of Vitamin D Levels

Deciding which vitamin D level to measure depends on what needs to be assessed clinically. 25(OH)D, though metabolically inactive, is the major circulating from of vitamin D. It is generally the best indicator of overall vitamin D status and is used to correlate vitamin D stores with clinical disease. The circulating half-life is 2 to 3 weeks (Table 1). 1,25(OH)_2_D, the metabolically active form, is closely regulated by 25(OH)D, PTH, calcium and phosphorus, and is measured to assess calcium metabolic disorders related to the renal production of 1,25(OH)_2_D. Its circulating half-life is 4 to 6 hours.

Low 25 (OH)D levels lead to decreased intestinal absorption of calcium causing a transient decrease of ionized calcium. This signals an increase of PTH to mobilize calcium from bones, increase tubular reabsorption of calcium from the kidneys, and increase production of 1,25(OH)_2_D by the kidneys. This increased production of 1,25(OH)D_2_D may not always need to occur, therefore low 25(OH)D may be associated with normal or elevated 1,25(OH)_2_D levels [4].

**Table 1:** Current accepted definitions of various Vitamin D levels include: [9].

>150 ng/mL	Toxicity
100 ng/mL	Maximum upper limit
40–70 ng/mL	Ideal range
>30 ng/mL	Sufficient
21–29 ng/mL	Insufficient
<20 ng/mL	Deficient

Vitamin D intoxication is defined as a 25(OH)D level >150 ng/mL associated with hypercalcemia, hypercalciuria, and hyperphosphatemia. Sunlight destroys excess vitamin D that is produced in the body so it is not possible to get vitamin D intoxication from sun exposure alone. Studies on outdoor workers during summer months found naturally produced vitamin D levels averaged around 50 ng/mL. Lifeguards at the beach had reported levels of 100 to 125 ng/mL without evidence of toxicity [10]. Toxicity could potentially occur with supplementation with vitamin D of greater than 10,000 IU daily over a prolonged period of time.

Vitamin D intoxication did occur in children who received an erroneously manufactured dietary supplement of fish oil with added vitamin D. These children presented with symptoms of hypercalcemia—weakness, constipation, loss of appetite, nausea, vomiting—and found to have serum calcium levels of 13.4 to 18.8 mg/dL. They also had measured levels of 25(OH)D of 340 to 962 ng/mL. When supplement intake was discovered and the product tested, the vitamin D was 4,000 times its stated amount. Estimated intakes of vitamin D for these children were between 266,000 to 800,000 IU per day. With discontinuation of the dietary supplement and with treatment, calcium levels normalized within 3 days and 25(OH)D levels normalized within 2–3 months [11].

On the other hand, insufficient and deficient levels, <30 ng/mL and <20 ng/mL respectfully, are commonly found in the general population. A recent National Health and Nutrition Examination Survey (NHANES) estimated that 10.3% of US children aged 6–18 years (population estimate 5.5 million) have 25(OH)D levels <16 ng/mL. Generally, vitamin D levels were lowest in African-American children averaging 20 ng/mL and Hispanics at 24 ng/mL. Most of these children showed some evidence of bone demineralization on standard radiographs [12,13]. It is unclear if this was related specifically to skin pigment, diet, or lifestyle or combination thereof.

Only 10% to 15% of dietary calcium and 60% of dietary phosphorus is absorbed from the intestinal tract in low vitamin D states. When 25(OH)D levels fall below 40 ng/mL, PTH is activated due to the decrease in calcium absorption from the intestines. PTH activates osteoblasts that stimulate the formation of osteoclasts which dissolve the calcium: phosphorus collagen matrix in bone. If not remedied, this can lead to osteopenia and rickets. The incidence of rickets in the industrialized world has increased over the past two decades as documented in the US, Canada, and Australia [14,15,16]. Rickets is most prevalent in darker pigmented races (immigrant refugees), in those living at higher latitudes, and in breast or formula fed babies who do not receive adequate vitamin D supplementation.

**Figure 2:**
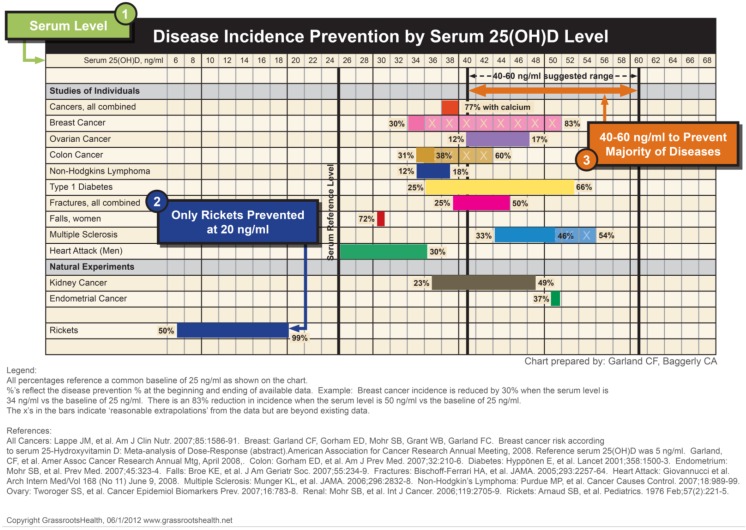
Disease Incidence Prevention by serum 25(OH)D levels. [18] (Used with permission).

Vitamin D experts advocate targeting 25(OH)D levels of 40 to 70 ng/mL to achieve the optimal skeletal function without toxicity [3,9]. Maintaining 25(OH)D levels above 40 ng/mL keeps PTH suppressed and allows for most favorable absorption of calcium from the intestines. Beyond the skeletal benefits, studies are now showing a correlation of improved health outcomes with higher levels of vitamin D. Advocating for vitamin D levels to reach the optimal levels noted above has been controversial and there have been conflicting recommendations since the release of the 2010 IOM report on vitamin D supplementation. This report stated that the recommended daily allowance (RDA) of 600 IU is the upper limit that should be given to any child or adult regardless of measured blood levels [17]. Based on pooled published studies, however, primarily in adults, the disease incidence prevention from higher 25(OH)D levels was significant. This could have important implications in preventative health care for children Figure 2 [18].

### 2.3. Extraskeletal Effects of Vitamin D

Vitamin D is known to have direct effects on innate immunity. Vitamin D receptors (VDR) are present in lymphocytes, monocytes and macrophages. In TB infections, for example, immune cells up-regulate their own expression of VDR and 1-α-hydroxylase increasing the local production of 1,25(OH)_2_D. This activates the transcription of antimicrobial peptides, cathelicidins and defensins [6]. When serum 25(OH)D levels fall below 20 ng/mL, monocytes and macrophages cannot initiate the innate immune response [19]. This activation of antimicrobial peptides also occurs in the skin and epithelial cells throughout the body. Localized injury or insult of the mucocutaneous barrier by viruses or bacteria activates 1-α-hydroxylase in tissues thereby increasing the local production of 1,25(OH)_2_D. This augments the expression of tissue cathelicidins and defensins thereby producing antimicrombial effects at the site of insult. The fact that the incidence of upper respiratory illnesses and influenza is higher after the winter solstice, in higher latitudes, in children with rickets, and in the institutionalized again seems to support the theory that the lack of vitamin D increases susceptibility to illness. In a study on vitamin D and osteoporosis in adults, those participants with low vitamin D levels were 40% more likely to report a recent URI compared to those whose 25(OH)D levels were > 30 ng/mL [20].

Prospective trials have been done studying the effects of vitamin D on the incidence of microbial illness in children. One study found an inverse association between cord-blood levels of 25 (OH)D and the risk of developing infections in infants. Newborns with 25(OH)D levels of <10 ng/mL were more likely to develop upper respiratory infections or otitis media by 3 months of age and wheezing at 15 months compared to those who had higher levels [21]. Cord blood vitamin D deficiency in otherwise healthy neonates was also associated with an increased risk of developing respiratory syncytial virus (RSV) during infancy. Neonates born with 25(OH)D concentrations <20 ng/mL had a six fold increased risk of developing a lower respiratory tract infection with RSV in the first year of life compared with those with 25(OH)D concentrations ≥30 ng/mL [22]. A randomized controlled trial compared the use of 1200 IU of vitamin D to placebo in 334 Japanese children during the months of December through March on the incidence of influenza A. The outcomes showed the study group was 58% less likely to get influenza A compared to the placebo group. Additionally, of the children who also had asthma, only 2 in the study group *vs.* 12 in the placebo group contracted influenza A [23]. 

Vitamin D also plays a role in adaptive immunity with nuclear VDR and vitamin D-activating enzymes present in both T and B cells. In the presence of vitamin D, T cells inhibit the secretion of pro-inflammatory Th-1 cytokines (IL-2, interferon γ, TNF-α) and promotes the production of the more anti-inflammatory Th-2 cytokines (IL-3, 4, 5, 10). Vitamin D also controls B-cell activation and proliferation thus reducing the production of auto-reactive antibodies [24]. This is important as epidemiological studies have shown an association between vitamin D deficiency and the increased incidence of autoimmune diseases. Many of these diseases, such as multiple sclerosis (MS), rheumatoid arthritis (RA), Crohn’s disease, and Type 1 diabetes mellitus (DM), are more prevalent in populations residing in higher latitudes. Women living at 35° N latitude for the first 10 years of life had an almost 100% increased risk of developing MS [25]. Women at this same latitude who took >400 IU of Vitamin D daily had a 42% decreased risk of developing MS [26]. Ten thousand three hundred sixty-six children in Finland received 2,000 IU of Vitamin D daily for the first year of life and then followed for the next 31 years. These children had a 78% reduced risk of developing Type 1 DM compared to those who did not receive this supplementation [27].

It is suggested that both MS and Type 1 DM may be caused by viral infections early in life. These infections trigger the destruction of myelin (in MS) and islet cells (in DM) from the action of excess pro-inflammatory cytokines and autoantibodies found in low vitamin D states. Increasing vitamin D intake during pregnancy reduced the development of islet cell auto-antibodies in offspring, thus supporting aspects of this theory [28].

The benefit of adequate vitamin D has also been demonstrated in children with asthma, eczema, and allergies. One thousand ninety-four mother-child pairs were evaluated for vitamin D levels and the incidence of childhood wheezing. Mothers with the highest intake of vitamin D were 61% less likely to have a child with recurrent wheeze, and for each 100 IU increase of intake, the risk fell by 19% [29].

A RCT compared the use of budesonide alone to budesonide plus daily supplementation with vitamin D 500 IU in children newly diagnosed with asthma. At six months, both groups had significant improvements in lung function as measured by FEV-1 and the Asthma Therapy Assessment Questionnaire (ATAQ). There were significantly fewer asthma exacerbations, however, in the vitamin D group compared to the budesonide only group. It was noted that each exacerbation was precede by an acute respiratory infection thereby it was surmised that vitamin D intake reduced the incidence of acute URI that triggered asthma exacerbations [30].

Children with eczema who took vitamin D 4000 IU daily for 21 days had significant increases in cathelecidin levels and reduced colonization of skin pathogens [31]. Low vitamin D levels correlated with sensitivity to 11 of 17 allergens tested via IgE RAST for food and environmental triggers. Children with 25(OH)D levels <15 ng/mL were more likely to have peanut, ragweed and oak allergy [32].

### 2.4. Vitamin D and Cancer

Practically all tissues express 1-α-hydroxylase that allows for local production of 1,25(OH)_2_D. This, in turn controls the local expression of genes that regulate cell proliferation and differentiation. It is known that activated vitamin D blocks cells at the G-1 phase, modulates production of several pro-oncogenes, and promotes apoptosis in some forms of cancer. Activated vitamin D also decreases angiogensis which reduces the risk of dissemination [33].

The majority of studies evaluating the association of vitamin D levels to cancer found a protective relationship between sufficient vitamin D status and lower risk of cancer [34]. People living at higher latitudes show an increase risk for developing Hodgkin’s lymphoma, pancreatic, breast, colon, and ovarian cancers. When compared to patients with these same diseases living at lower latitudes, those at higher latitudes were more likely to die, even when controlled for lifestyle. These statistics were associated with 25(OH)D levels <20 ng/mL [35]. In another epidemiological study, children and young adults exposed to the most sunlight had a 40% reduced risk of developing non-Hodgkin’s lymphoma [36].

Overall there is a strong inverse correlation for solar UVB irradiance and the incidence of various cancers—bladder, breast, cervical, colon, endometrial, esophageal, gastric, lung, ovarian, pancreatic, rectal, renal, vulvar, and Hodgkin’s and non-Hodgkin’s lymphoma. This same effect was not found with vitamin D oral supplementation [37].

The campaign for use of sun-screens and for sun avoidance was initiated to reduce the incidence of skin cancers. Though this has decreased some forms of non-melanoma cancer, the actual rate of melanoma has increased [38]. The working theory to explain this phenomenon suggests that sunscreens primarily block UVB rays to prevent sunburn, but do not fully block UVA or UVC rays that penetrate the deeper layers of skin causing DNA damage. With the reduced risk of sun burn, individuals often have prolonged sun exposures not realizing that deeper damage is occurring.

### 2.5. Vitamin D and Pain

Vitamin D increases muscle protein synthesis, possibly by activating second messengers and phosphorylation [39]. In mouse models, skeletal muscle hypersensitivity with increased numbers of nociceptor axions were documented in mice with vitamin D deficient states but not in those with vitamin D sufficient states [40]. Vitamin D deficiency has been associated with muscle pain and proximal muscle weakness with reports of heaviness in the legs, rapid fatigue, and problems with climbing stairs or mobility. Vitamin D deficiency is also frequently documented in patients diagnosed with fibromyalgia and non-specific musculoskeletal pain [41]. In pediatrics, vitamin D deficiency may present as atypical muscular pain and is found more typically in those who are Caucasian, have a history of being breastfed, are protected from the sun, and are obese [42].

In a recent clinical study of individuals with chronic pain, 71% of enrolled subjects were vitamin D deficient (<20 ng/mL) and 21% were vitamin D insufficient (20–30 ng/mL). Only 8% of these subjects had levels > 30 ng/mL. Lower vitamin D levels were significantly associated with higher pain sensitivity scores [43]. In another study, resolution of pain occurred when subjects with chronic pain were adequately supplemented with vitamin D-3 to reach 25-hydroxy Vitamin D levels of >30 ng/mL [44].

Very low vitamin D levels have been found in a survey of children with migraine as well [45]. Vitamin D deficiency is associated with higher levels of pro-inflammatory and pro-coagulatory biomarkers—some of the same biomarkers known to be elevated in adult and pediatric patients with migraine. These biomarkers are indicative of endothelial activation and vascular reactivity caused by inflammation and oxidative stress [46,47]. Vitamin D, because of its immune modulation, nerve stabilizing, and antithrombotic effects may decrease the incidence of migraine. Thus far no randomized, placebo controlled studies have been conducted in adults or children using vitamin D to prevent or treat migraine, however several case reports suggest that vitamin D may be effective in reducing migraine pain in adults. There was demonstrated reduction of headache pain when patients were supplemented with vitamin D (1000–1500 IU) and calcium (1000–1500 mg). Though the role of calcium in suppressing pain could not be ruled out, vitamin D seemingly was more vital for headache relief. Serum calcium levels normalized within the first week of therapy, but headache pain only abated once there was normalization of the 25(OH)D levels which occurred at 4 to 6 weeks of treatment [48].

### 2.6. Vitamin D and Mental Health

Vitamin D is emerging as a steroid derivative with neuroactive properties that have direct effects on brain development. There is a wide distribution of VDR and 1-α-hydroxylase throughout the brain allowing for local production of activated vitamin D. 1,25(OH)_2_D regulates nerve growth factor and glial cell line-derived neurotropic factor which orchestrates the cellular architecture of the brain. Activated vitamin D also has neuroprotective effects via neuromodulation, anti-inflammatory, anti-ischemic, and anti-oxidant properties [6].

Having adequate vitamin D levels in-utero and early stages of life ensures normal receptor transcriptional activity vital for brain development and mental functioning [49]. Vitamin D affects the proteins directly involved in learning, memory, motor control, and social behavior [50], and is closely associated with executive functioning such as goal-directed behavior, attention, and adaptability to change [51].

Vitamin D deficiency and insufficiency are found frequently in adolescents with severe mental illness. Vitamin D deficiency has been linked to an increased risk of developing schizophrenia. In one study of adolescents admitted to an acute mental health facility, those who were vitamin D deficient were 3½ times more likely to have psychotic features when compared to vitamin D sufficient patients [52]. In another review of psychiatric patients, those with the lowest levels of vitamin D (<20 ng/mL) were more likely to be male, of Middle East, South-East Asian, or African ethnicity, and had diagnoses of autism and schizophrenia. When supplemented with 1000 to 4000 IU of vitamin D, many had clinical improvement [53].

Vitamin D deficiency has also been associated with depression and seasonal effective disorder. Vitamin D deficiency decreases the expression of the enzyme catechol-O-methyl transferase (COMT), required for dopamine and serotonin metabolism. This has been associated with negative CNS effects in animal studies [50]. In human studies, resolution of depression occurred when depressed adolescents were adequately supplemented with vitamin D-3 to reach 25(OH)D levels of >30 ng/mL [54].

There has been an undeniable increase in the incidence of autism since the 1980s that is not explained by changes in diagnostic criteria or changes in reporting. The incidence of autism in 1980 was approximately 1:200 but now stands at 1:68 in the US [55]. Studies in Asia, Europe, and North America have identified individuals with autism with an average prevalence of about 1%. A study in South Korea reported a prevalence of 2.6% [56]. Current research suggests that autism may have an underlying genetic susceptibility with biochemical abnormalities. This susceptible state is impacted by environmental influences which trigger the development of autism through mitochondrial dysfunction, immune dysregulation, inflammation, oxidative stress, methylation problems, and toxicity [57].

The lack of adequate vitamin D, in theory, may play a role in the development of autism (Table 2). What is currently known:

**Table 2:** Commonalities between Autism and Vitamin D deficiency.

Autism	Vitamin D
Serotonin, which promotes social behavior and facilitates accurate assessment of emotional social cues, is reduced in autistic brains. Low Vitamin D levels are also commonly found in autism.	1,25(OH)_2_D activates the transcription of tryptophan hydroxylase-2, an enzyme that converts tryptophan to serotonin in the brain [58].
Increased levels of inflammatory cytokines are found in autism-- IL-1β, IL-6, IL-8, *etc* [59]	Inflammatory cytokines (IL-6, IL-10, CRP, *etc*) are elevated in Vitamin D deficiency [60].
Low glutathione levels found in autism—difficulty excreting heavy metals [61].	Vitamin D increases glutathione in the brain—suggesting a role for the hormone in brain detoxification pathways [62].
Depakote has been associated with autism in children of mothers taking this during pregnancy [63].	Depakote lowers Vitamin D levels [64].
Seizures are common in children with autism [65].	Normalization of serum vitamin 25(OH)D level has an anticonvulsant effect [66].
Autism occurs more frequently in male > females.	Estrogen protects the developing female brain from Vitamin D deficiency (oxidative stress). Testosterone does not [67].

Also known is the strong correlation between latitude and the incidence of autism, increased number of children with autism born during the winter months, more autistic children are born to multiparous women, higher prevalence of autism in geographic areas with highest cloud cover and precipitation, and greater incidence in urban* vs.* rural populations (possibly from air pollution, tall buildings, indoor living) [68,69,70,71]. The vitamin D theory does not diminish the genetic or other environmental contributions; rather it may allow the genetic tendency of autism to express itself in a state of vitamin D deficiency.

### 2.7. Vitamin D in Pregnancy and Effects on Fetus and Newborn

Vitamin D deficiency and insufficiency occur in 27% to 91% of all pregnant women depending on the country of residence [7]. A meta-analysis of studies assessing vitamin D in pregnancy showed an inverse association of low vitamin D levels and the risk of pre-eclampsia, gestational diabetes, pre-term births, and small for gestational age babies [72]. Low maternal vitamin D predisposes the fetus/newborn to low vitamin D stores as well leading to rickets, wheezing, and upper respiratory tract infections and mental health issues as noted before. Vitamin D deficiency in pregnancy has also been associated with the increased development of IgE-specific allergens and eczema in offspring [73,74]. An Australian study found maternal vitamin D insufficiency during pregnancy was associated with language impairment in their offspring [75]. Yet another study found inadequate vitamin D during pregnancy effected tooth calcification in the newborn. Women who had the lowest 25 (OH)D levels had children with a greater incidence of tooth enamel hypoplasia and early childhood caries by age 1 year [76].

Exclusive breastfeeding without adequate sun exposure or vitamin D supplementation contributes to ongoing vitamin D deficiency in newborns as unsupplemented breast milk only contains 20 to 80 IU/L of vitamin D. (By comparison, infant formula is required to be fortified with 40 to 100 IU of vitamin D per 100 kcal to give approximately 270 to 677 IU/L.) Research revealed that lactating women often need 25 (OH)D levels of 40 to 50 ng/mL to provide sufficient vitamin D in breast milk for nursing infants [77]. Daily doses of 4000 to 5000 IU daily of cholecalciferol (Vitamin D-3) were required to achieve optimal 25(OH)D serum levels in lactating women and did not cause toxicity [78,79]. This dose is much higher than the recommended daily allowance of 600 IU suggested by the IOM for adults and lactating women and also significantly higher than the amount of vitamin D found in standard pre-natal or multivitamins which typically provide 400 to 600 IU per dose.

### 2.8. Recommended Supplementation

Being aware of the risk factors associated with the development of vitamin D deficiency will help guide clinicians to both assess for and intervene with supplementation as needed.

• Infants who are exclusively breast fed or ingest less than 1000 mL of infant formula a day
• Children living north or south of the 33° latitudes or in urban/polluted environments
• Those that are obese, deeply pigmented or, for cultural reasons, cover their skin with clothing
• Those with disorders of digestion or who are on medications that prevents absorption of vitamin D
• Children who are institutionalized, hospitalized, or attend schools that limit outside play

One can choose to measure 25(OH)D levels to document vitamin D deficiency, but with the widespread findings of insufficiency and deficiency in most cultures, it is relatively safe to start vitamin D supplementation without this information.

Supplementing with natural sunlight 15 to 30 min a day during the hours of 10 AM to 3 PM is optimal for prevention and treatment of vitamin D deficiency; however, this may not be available to all those at risk. Vitamin D-3 (cholecalciferol) or vitamin D-2 (ergocalciferol) are both available for oral supplementation and both are transformed in the liver and kidneys to the active 1,25(OH)_2_D. Research has found, however, that Vitamin D3 is approximately 87 percent more potent in raising and maintaining vitamin D concentrations and produces a 2- to 3-fold greater storage of vitamin D than does vitamin D2 [80]. 

Supplementing 400 IU daily of Vitamin D3, as recommended by the American Academy of Pediatrics (AAP) and the IOM, or up to 800 IU as recommended by the Canadian Pediatric Society may provide sufficient vitamin D to prevent rickets, but higher doses may be needed to achieve other health benefits [81]. For every 100 IU intake of vitamin D3, serum levels increase by 1 ng/mL when given over 3 to 4 months [82]. For a child who is deficient, a standard dose of 400 IU will not correct this problem. A study of breast fed infants found that only a dose of 1600 IU/day (and not 400, 800, or 1200 IU) could raise 25(OH)D to a level >28 ng/mL after 3 months [83]. It is recommended by experts in the field of vitamin D research that healthy children receive approximately 1000 IU per 11 Kg of body weight each day to achieve optimal 25(OH)D levels year round [84]. Having a baseline 25(OH)D level can help guide initial dosing with a follow- up level in 2 to 3 months to monitor efficacy.

### 2.9. Potential Economic Impact

Given the high incidence of vitamin D insufficiency/deficiency, convincing evidence for benefit, and the strong evidence of safety, vitamin D supplementation should be utilized more now than ever. Interest groups and researchers have been advocating for education on vitamin D deficiency and for wide-spread vitamin D supplementation to reduce the burden of disease. Despite these efforts, vitamin D deficiency and its sequellae persist [85]. 

Grant *et al.* assessed the economic burden and premature death related to vitamin D deficiency in North America and estimated what would change if 25(OH)D levels were optimized in all individuals. They used data from studies that demonstrated significant vitamin D effects on disease incidence or mortality rates. From these studies, they estimated that all-cancer incidence rates would decrease approximately 25%, influenza and pneumonia rates would decrease approximately 30%, septicemia would decrease 25%, multiple sclerosis by 40%, and negative pregnancy outcomes (including asthma, infections, bone disorders, heart failure, and autism in the offspring) would be reduced by 10% [86]. In assessing the overall cost savings in Canada alone, they estimated the death rate would fall by 16% and that the total economic burden would decrease by 6.9% or approximately $14.4 billion per year.

Estimated annual costs of various diseases influenced by vitamin D in the United States are listed below (Table 3).

**Table 3:** Estimated annual costs of common disorders that potentially could be attenuated by adequate Vitamin D levels.

Disease	Direct (in US Dollars)	Indirect (in US Dollars)	Total Annual Expense (US)
Influenza	10.4 billion	16.3 billion	26.7 billion [87]
Asthma	50.1 billion	5.9 billion	56 billion [88]
Upper respiratory infections	17 billion	23.5 billion	40 billion [89]
Autism	__	__	126 billion [90]
Cancer	86.6 billion	130 billion	216.6 billion [91]

If one used the same data from Grant’s study, the saving of life and money could be even greater in the United States which has almost 10 times the population of Canada. With a current population near 320 million, supplementing each person in the US with 3000 IU per day of vitamin D3 to achieve optimal 25(OH)D levels would cost approximately $3 billion per year (based on current retail costs found on the Internet of approximately $10 per year). This relatively low cost intervention could potentially save $84 billion dollars in total health care costs just for these diseases alone.

## 3. Conclusions

Beyond the skeletal effects it pro-offers, there is more evidence now supporting the beneficial benefits vitamin D has on immune health, mental health, and overall life expectancy. For the sake of public health, instituting a proactive program, much like the current immunization programs, using vitamin D supplementation could have dramatic impact on overall health worldwide by reducing the physical, emotional, and economic burden of disease.
